# Supplementary material for: Content-rich biological network constructed by mining PubMed abstracts
Source: BMC Bioinformatics. 2004 Oct 8;5:147. doi: 10.1186/1471-2105-5-147 (PMC528731; doi:10.1186/1471-2105-5-147)
Supplement: Additional File 5 — The original Chilibot query results of the term "long-term potentiation (LTP)" and 22 other terms, limiting the latest references analyzed to the years 1990, 1995, 2000, and 2004. [file 1471-2105-5-147-S5.bz2 › chilibotAdditionalFile5/ltp2004/html/PLC_NMDA.html]

 


 **PLC** and **NMDA** 
  
Found 19 abstracts in PubMed,  **19 abstracts were retrieved and analyzed**.  


---

 Search Google  |
 PDF files only 
|  EDU domain only 

---

**Interactive relationship** (e.g. stimulation, inhibition, etc)

- mGluR1alpha mediated potentiation of  **NMDA**  responses was eliminated by the  **PLC**  inhibitor U 73122.  Ref: 11378156 Neuropharmacology, 2001
- **PLC**  beta 1 TC mutants develop a loss of  **NMDA**  receptors in the stratum oriens of region CA1, apoptotic neuronal death, and reduced hippocampal PKC activity.  Ref: 12504592 Mol Cell Neurosci, 2002
- Both glutamate induced neuronal degeneration and the increases in  **PLC**  delta immunoreactivity were prevented by removal of extracellular calcium or the application of an N methyl D aspartate  **NMDA**  receptor antagonist, MK 801.  Ref: 7563235 J Neurosci Res, 1995
- Both kinetic analysis and equilibrium saturation experiments indicated that  **PLC**  treatment produced a decrease in affinity for 3H N 1 thienyl cyclohexyl piperidine 3H TCP, a ligand for the N methyl D aspartate  **NMDA**  receptor associated ionic channel, when the channel was fully activated by high concentrations of glutamate and glycine but increased its binding under conditions in which the channel was presumably closed.  Ref: 2154675 Mol Pharmacol, 1990
- It was revealed that nitric oxide NO formation secondary to calcium influx by N methyl D aspartate  **NMDA**  receptor activation leads to modifications of  **PLC**  delta 1 similar to those seen in AD brains.  Ref: 8534418 Alzheimer Dis Assoc Disord, 1995
- These results suggest that NO formation secondary to calcium influx by  **NMDA**  receptor activation leads to similar modifications of  **PLC**  delta to those seen in AD.  Ref: 7563235 J Neurosci Res, 1995
- These results demonstrate that BDNF functions at the spinal level to enhance synaptic efficacy in an  **NMDA**  receptor dependent manner and requires the action of the  **PLC**  PKC pathway.  Ref: 14622147 Eur J Neurosci, 2003
- The results demonstrate that  **NMDA**  receptor subunits NR2A and NR2B will bind to the SH2 domains of  **PLC**  gamma.  Ref: 9231720 J Neurochem, 1997
- In contrast to  **PLC**  from C. perfringens, phosphatidylinositol specific  **PLC**  treatment did not detectably modify the binding properties of the quisqualate AMPA receptor or the  **NMDA**  receptor channel.  Ref: 2154675 Mol Pharmacol, 1990
- These results underlie the role of  **NMDA**  receptors in the early stage of consolidation of a simple odor reward associative memory and confirm the role of the  **PLC**  in the consolidation of long term memory.  Ref: 12843246 J Neurosci, 2003

**Parallel relationship** (e.g. studied together, co-existance, homology, etc.)

- Binding of 3H glutamate and 3H glycine to the  **NMDA**  receptors was not modified by  **PLC**  treatment, but there was a large decrease in the binding of the  **NMDA**  antagonist 3H 3 2 carboxypiperazine 4 yl propyl 1 phosphonic acid.  Ref: 2154675 Mol Pharmacol, 1990
- Thus, Group I mGluRs potentiate  **NMDA**  responses via activation of  **PLC** .  Ref: 11378156 Neuropharmacology, 2001
- This article has focused narrowly on specific cellular elements of the stress adaptational mechanisms, including the AC PKA and  **PLC**  PKC transductional cascades, together with specific response elements, such as the HPA axis, BDNF, and  **NMDA**  receptors.  Ref: 11147243 Psychiatr Clin North Am, 2000
- Both  **NMDA**  and KA attenuated PI hydrolysis evoked by the muscarinic receptor agonist carbachol about 30%, indicating that the activation of iGluRs exerts a relatively general inhibitory effect on the function of different  **PLC**  coupled metabotropic receptors.  Ref: 9759922 Neurochem Int, 1998
- Blockade of  **NMDA**  receptors into the  **PLC**  but not into the hippocampus impaired memory formation of the odor reward association.  Ref: 12843246 J Neurosci, 2003
